# Learning from the past: did experience with previous epidemics help mitigate the impact of COVID-19 among spine surgeons worldwide?

**DOI:** 10.1007/s00586-020-06477-6

**Published:** 2020-06-04

**Authors:** Joseph A. Weiner, Peter R. Swiatek, Daniel J. Johnson, Philip K. Louie, Garrett K. Harada, Michael H. McCarthy, Niccole Germscheid, Jason P. Y. Cheung, Marko H. Neva, Mohammad El-Sharkawi, Marcelo Valacco, Daniel M. Sciubba, Norman B. Chutken, Howard S. An, Dino Samartzis

**Affiliations:** 1 Department of Orthopaedic Surgery, Northwestern University, Chicago, IL USA; 2 Department of Orthopaedic Surgery, Hospital for Special Surgery, New York, NY USA; 3 Department of Orthopaedic Surgery, Rush University Medical Center, Orthopaedic Building, Suite 204-G, 1611 W Harrison Street, Chicago, IL 60612 USA; 4 The International Spine Research and Innovation Initiative, Rush University Medical Center, Chicago, IL USA; 5 Research Department, AO Spine International, Davos, Switzerland; 6 Department of Orthopaedics and Traumatology, The University of Hong Kong, Hong Kong, China; 7 Department of Orthopaedic and Trauma Surgery, Tampere University Hospital, Tampere, Finland; 8 Department of Orthopaedic and Trauma Surgery, Assiut University Medical School, Assiut, Egypt; 9 Department of Orthopaedics, Churruca Hospital de Buenos Aires, Buenos Aires, Argentina; 10 Department of Neurosurgery, John Hopkins University, Baltimore, MD USA; 11 Department of Orthopaedic Surgery, University of Arizona College of Medicine, Phoenix, AZ USA

**Keywords:** COVID-19, Coronavirus, Spine, Surgeons, Worldwide, Global, Impact

## Abstract

**Purpose:**

Spine surgeons around the world have been universally impacted by COVID-19. The current study addressed whether prior experience with disease epidemics among the spine surgeon community had an impact on preparedness and response toward COVID-19.

**Methods:**

A 73-item survey was distributed to spine surgeons worldwide via AO Spine. Questions focused on: demographics, COVID-19 preparedness, response, and impact. Respondents with and without prior epidemic experience (e.g., SARS, H1NI, MERS) were assessed on preparedness and response via univariate and multivariate modeling. Results of the survey were compared against the Global Health Security Index.

**Results:**

Totally, 902 surgeons from 7 global regions completed the survey. 24.2% of respondents had prior experience with global health crises. Only 49.6% reported adequate access to personal protective equipment. There were no differences in preparedness reported by respondents with prior epidemic exposure. Government and hospital responses were fairly consistent around the world. Prior epidemic experience did not impact the presence of preparedness guidelines. There were subtle differences in sources of stress, coping strategies, performance of elective surgeries, and impact on income driven by prior epidemic exposure. 94.7% expressed a need for formal, international guidelines to help mitigate the impact of the current and future pandemics.

**Conclusions:**

This is the first study to note that prior experience with infectious disease crises did not appear to help spine surgeons prepare for the current COVID-19 pandemic. Based on survey results, the GHSI was not an effective measure of COVID-19 preparedness. Formal international guidelines for crisis preparedness are needed to mitigate future pandemics.

## Introduction

The COVID-19 pandemic has rapidly become one of the most catastrophic global health crises of our time [1–3]. Patients infected with COVID-19 have placed an enormous strain on healthcare systems across the world in both the ambulatory and inpatient settings [4]. Many initial epidemiologic models predicted tremendous demands on existing hospital resources and staff across the globe [5–8]. Unfortunately, the ability to meet these demands has been variable around the world [9]. Some conjecture exists that this is due, in part, to different degrees of preparedness to treat and prevent spread of the virus [9]. For example, many countries have dealt with prior serious public health outbreaks, such as Severe Acute Respiratory Syndrome (SARS), Middle East Respiratory Syndrome (MERS), H1N1 Swine Flu, or Ebola [10–13]. The World Health Organization (WHO) and the global health community have made pandemic preparedness one of their main missions [14, 15], and research on pandemic preparedness is plentiful [9, 16–18].

In 2019, the Nuclear Threat Initiative (NTI) and the Johns Hopkins Center for Health Security (JHU) developed the Global Health Security Index (GHSI) [19]. It was the first comprehensive assessment of health security and pandemic preparedness across the 195 countries that make up the States Parties to the International Health Regulations (IHR 2005) [20]. The GHSI provided a ranking by overall pandemic preparedness, early detection capabilities, ability to mitigate a health disaster, along with numerous other variables. The goal of the GHSI project was to use data obtained from prior pandemics, along with information on international health systems, to spur measurable changes in global health security and improve the international capability to address infectious disease outbreaks [21].

While global pandemics are catastrophic events for the entire population, they are particularly impactful on healthcare systems. Resource limitations, healthcare worker illness, and severe economic repercussions have impacted providers and hospitals across the world. Previous studies have focused on the effect of COVID-19 on emergency room, critical care, and internal medicine specialties [22, 23]. However, the impact of preparedness on subspecialty surgical care, such as spine surgery, in the context of the COVID-19 pandemic is unknown [24–27]. With low back pain ranking as the most disabling condition worldwide and neck-related issues ranked as the fourth leading cause globally, there is a major demand for spine providers [28, 29]. Many spine surgeons have shifted away from their normal clinical duties to assist large multidisciplinary teams in caring for COVID patients [30]. A recent study by Louie et al. [27] highlighted, in over 900 spine surgeons worldwide, that COVID-19 had a substantial impact upon their patient care, practice, and personal lives; however, such impact varied. As such, it remains unknown whether previous experience with outbreaks/pandemics played a role in their preparedness, response, and perceptions. The current study addresses the role of prior infectious disease outbreaks on the preparedness, response, and impact of COVID-19 on spine surgeons across the world. This study also assessed the ability of the GHSI to predict preparedness and response to COVID-19.

## Methods

### Study design

The AO Spine COVID-19 and Spine Surgeon Global Impact Survey was developed by a working group of board-certified spine surgeons, epidemiologists, and statisticians who are experts in spinal disorders and represented different global regions. Question selection was based on a Delphi methodology [31, 32] to achieve consensus through several rounds of expert review before finalization. Overall scope of the survey included surgeon demographics, country and region of practice, COVID-19 perceptions, institutional preparedness and response, personal and practice impact, and future perceptions. Demographics obtained included country of practice, region of practice, population of city of practice, specialty, fellowship experience, year in practice, and practice type. Previous experience with SARS, MERS, H1N1, or Ebola was queried to ascertain experience with prior infectious disease outbreaks. Additional details of the survey can be found in the Louie et al. [27] report.

### Survey distribution

The 73-item survey was presented in English and distributed via email to the AO Spine membership who agreed to receive surveys for academic purposes (*n* = 3805). AO Spine represents the largest society composed of spine surgeons worldwide (www.aospine.org). Each recipient was instructed that they had nine days to complete the survey (March 27, 2020, to April 4, 2020). For all survey respondents, participation was voluntary, that they could end their participation at any time point, their involvement would be anonymous, and all data would be kept confidential. Participants were also informed that study findings would be disseminated in peer-reviewed journals, Web sites, and on social media platforms.

### Statistical analyses

All statistical analyses were performed with SAS (SAS Institute, Cary, NC). Graphical representation of survey responses was performed using RStudio v1.2.1335 (RStudio Inc, Boston, MA) and Excel (Microsoft Inc, Redmond, WA). Percentages and means were made for count data and rank-order questions, respectively. All means were presented with standard deviations (mean ± standard deviation). Statistical analyses were performed to assess significant differences in count data using a combination of Fisher’s exact and chi-squared tests, where applicable. Differences in continuous variables between groups were assessed using analysis of variance (ANOVA).

A nominal multivariate logistic regression was performed, controlling for baseline demographic differences between respondents with and without prior epidemic exposure, adjusting for covariates (e.g., home city population, geographic region, fellowship training, practice breakdown). Outcome variables with *p* < 0.200 on univariate analysis were assessed in the multivariate model. Variables with dichotomous categorical outcomes presented as odds ratios (OR) and 95% confidence intervals (CI) were also noted. An OR > 1 indicated increased occurrence of outcome with prior epidemic exposure. An OR < 1 indicated decreased occurrence of outcome (protective exposure). Variables with numerous categorical outcomes were presented as likelihood ratios. Linear regression analysis was performed to assess the relationship between GHSI and survey responses. *R*^2^ regression coefficients less than 0.3 were considered poor correlation [33–36]. The threshold for statistical significance for all tests was *p* < 0.05.

## Results

**Fig. 1:**
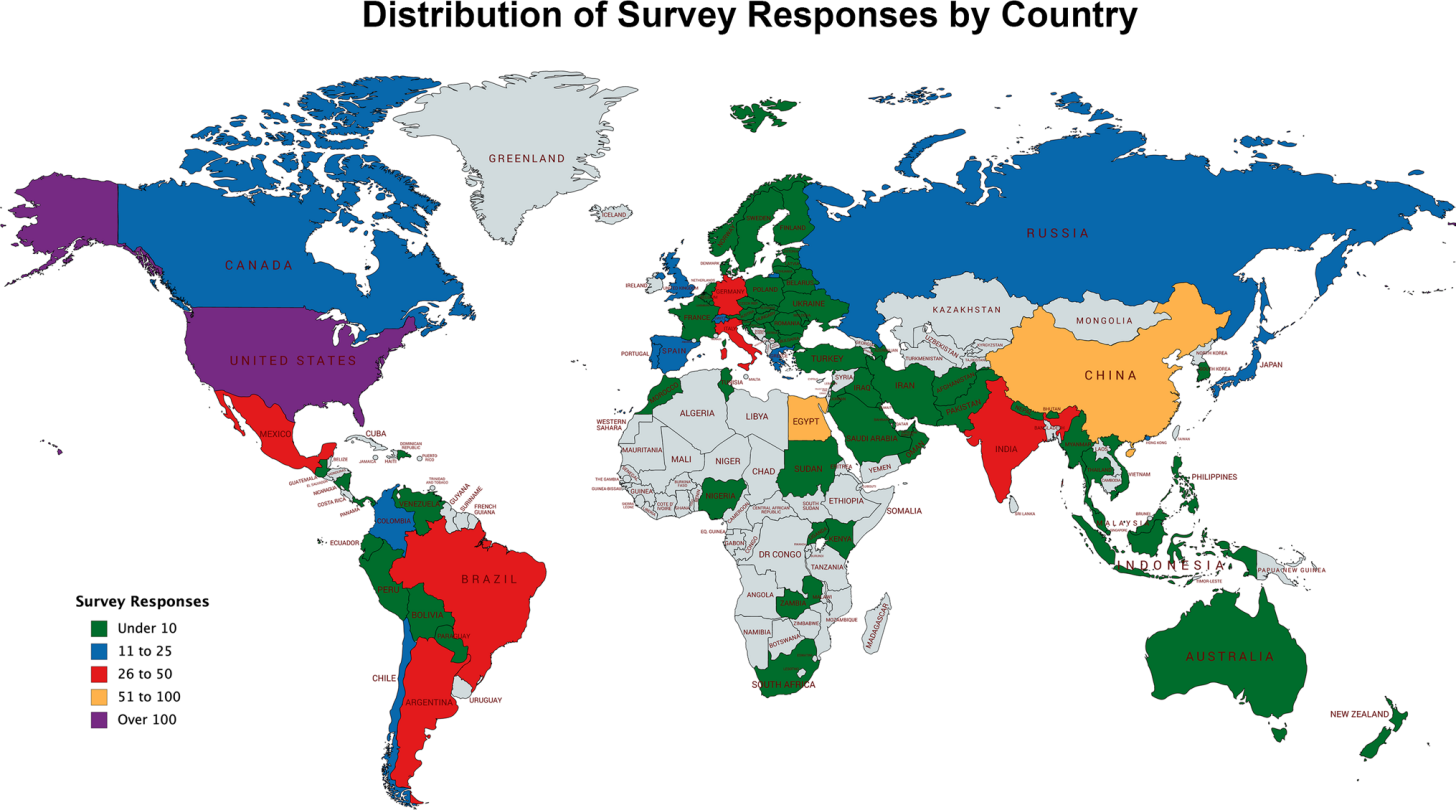
Distribution of survey responses by country. World map depicting number of survey responses received internationally. Color-filled countries indicate that at least one survey was received. Green, under 10 surveys received; Blue, 11 to 25; Red, 26 to 50; Orange, 51 to 100; Purple, over 100; Grey, no surveys received

In total, 902 spine surgeons responded to the survey, representing 91 distinct countries and 7 global regions (Africa, Asia, Australia, Europe, the Middle East, North America, and South America/Latin America). Of the 881 surgeons providing their region of practice, the greatest number of responses was from Europe (242/881; 27.5%), followed by Asia (213/881; 24.2%) and North America (152/881; 17.3%). Most survey responses were from the USA (128/902; 14.2%), China (73/902; 8.1%), and Egypt (66/902; 7.3%) (Fig. 1). A majority of respondents (647/902; 75.8%) reported no experience with recent epidemics (SARS, H1N1, MERS, or Ebola). The majority of respondents were male (826/881; 93.8%), aged from 35 to 44 years old (344/895; 38.4%), orthopedic surgeons (637/902; 70.6%), and primarily practiced in academic or private institutions (Table 1).

**Table 1:** Survey respondent demographics

	All respondents (*n* = 902)	Previous epidemic experience (%) (*n* = 255)	No previous epidemic experience (%) (*n* = 647)	*p* value
Age				0.5
25–34	130 (14.5)	31 (12.5)	99 (15.3)	
35–44	344 (38.4)	97 (39)	247 (38.2)	
45–54	245 (27.4)	77 (30.9)	168 (26)	
55–64	150 (16.8)	38 (15.3)	112 (17.3)	
65 +	26 (2.9)	6 (2.4)	20 (3.1)	
Sex				0.61
Female	55 (6.2)	17 (6.9)	38 (6.0)	
Male	826 (93.8)	229 (93.1)	597 (94.0)	
Estimated home city population				0.0003
< 100,000	46 (5.2)	12 (4.8)	34 (5.3)	
100,000–500,000	185 (20.7)	38 (15.3)	147 (22.8)	
500,000–1,000,000	136 (15.2)	28 (11.3)	108 (16.7)	
1,000,000–2,000,000	144 (16.2)	34 (13.7)	110 (17.1)	
> 2,000,000	382 (42.8)	136 (54.8)	246 (38.1)	
Geographic region				
Africa	44 (5.0)			< 0.0001
Asia	213 (24.2)			
Australia	8 (1.0)			
Europe	242 (27.5)			
Middle East	77 (8.7)			
North America	152 (17.3)			
South America/Latin America	145 (16.5)			
Previous epidemic experience				
None	647 (71.7)	0 (0)	647 (100)	< 0.0001
SARS	98 (10.9)	97 (38.0)	0 (0)	< 0.0001
H1N1 swine flu	128 (14.2)	127 (49.8)	0 (0)	< 0.0001
MERS	17 (1.9)	17 (6.7)	0 (0)	< 0.0001
Ebola	15 (1.7)	15 (5.9)	0 (0)	< 0.0001
Specialty				0.68
Neurosurgery	234 (26.4)	65 (26.5)	169 (26.4)	
Orthopedics	637 (72.0)	178 (72.7)	459 (71.7)	
Pediatric surgery	2 (0.2)	0 (0)	2 (0.3)	
Neurosurgery	12 (1.4)	2 (0.8)	169 (26.4)	
Fellowship trained	645 (71.5)	192 (75.3)	453 (70.0)	0.11
Years since training completion				0.43
Less than 5 years	161 (25.3)	49 (25.9)	112 (25.1)	
5–10 Years	141 (22.2)	41 (21.7)	100 (22.4)	
10–15 Years	104 (16.4)	38 (20.1)	66 (14.8)	
15–20 Years	117 (18.4)	29 (15.3)	88 (19.7)	
Over 20 Years	113 (17.8)	32 (16.9)	81 (18.1)	
Practice type				0.33
Academic	405 (45.4)	124 (50.4)	281 (43.5)	
Academic/private combined	204 (22.9)	51 (20.7)	153 (23.7)	
Private	144 (16.1)	36 (14.6)	108 (16.7)	
Public/local hospital	139 (15.6)	35 (14.2)	104 (16.1)	
Practice breakdown				
Percent research				0.037
0–25%	731 (81.9)	192 (77.1)	539 (83.7)	
26–50%	129 (14.4)	49 (19.7)	80 (12.4)	
51–75%	21 (2.4)	4 (1.6)	17 (2.6)	
76–100%	12 (1.3)	4 (1.6)	8 (1.2)	
Percent clinical				0.22
0–25%	22 (2.5)	6 (2.4)	16 (2.5)	
26–50%	87 (9.7)	25 (10.1)	62 (9.6)	
51–75%	194 (21.8)	65 (26.2)	129 (20.0)	
76–100%	590 (66.1)	152 (61.3)	438 (67.9)	
Percent teaching				
0–25%	668 (74.9)	6 (2.4)	16 (2.5)	
26–50%	152 (17.0)	25 (10.1)	62 (9.6)	
51–75%	50 (5.6)	65 (26.2)	129 (20.0)	
76–100%	22 (2.5)	152 (61.3)	438 (67.9)	

Respondents overall reported a moderate to high level of concern regarding the COVID-19 outbreak, with a mean score of 3.7 ± 1.2 on a scale of one to five. Recent epidemic experience did not impact mean worry (3.8 ± 1.1 vs. 3.7 ± 1.2, *p* = 0.400), but did increase the proportion of those reporting personal health as a main source of stress (47.8% vs 36.5%, *p* = 0.002). The three most common stressors identified for respondents with previous epidemic experience groups were family health (74.5%), personal health (47.8%), and economic issues (46.7%). The three most common stressors identified for respondents without previous epidemic experience groups were family health (69.6%), community health (42.5%), and timeline to resume work (42%) (Table 2).

**Table 2:** COVID-19 perceptions stratified by previous epidemic experience

	All respondents (*n* = 841)	Previous epidemic experience (%) (*n* = 255)	No previous epidemic experience (%) (*n* = 647)	*p* value
Mean worry (1—not worried to 5—very worried)	3.7 ± 1.2	3.8 ± 1.1	3.7 ± 1.2	0.4
3 Greatest stressors				
Personal health	358 (39.7)	122 (47.8)	236 (36.5)	0.0017
Family health	640 (71.0)	190 (74.5)	450 (69.6)	0.14
Community health	370 (41.0)	95 (37.3)	275 (42.5)	0.15
Hospital abilities	332 (39.0)	99 (38.8)	253 (39.1)	0.94
Timeline to resume work	378 (41.9)	106 (41.6)	272 (42.0)	0.9
Government/leadership	154 (17.0)	52 (20.4)	102 (15.8)	0.1
Return to nonessential activities	116 (12.9)	34 (13.3)	82 (12.7)	0.79
Economic issues	385 (42.7)	119 (46.7)	266 (41.1)	0.13
Currently coping w/the stress				
Exercise	463 (51.0)	131 (51.4)	332 (51.3)	0.99
Music	330 (36.6)	112 (43.9)	218 (33.7)	0.0041
Meditation/mindfulness	118 (13.0)	42 (16.5)	76 (11.8)	0.058
Tobacco	29 (3.2)	5 (2.0)	24 (3.7)	0.21
Alcohol	89 (9.9)	23 (9.0)	66 (10.2)	0.59
Research projects	244 (27.5)	76 (29.8)	168 (36)	0.24
Spending time w/family	578 (64.1)	162 (63.5)	416 (64.3)	0.83
Spiritual/religious activities	116 (12.9)	35 (13.7)	81 (12.5)	0.63
Reading	458 (50.8)	125 (49.0)	333 (51.5)	0.51
Television	394 (43.7)	101 (39.6)	293 (45.3)	0.12
Telecommunication with friends	322 (35.7)	92 (36.1)	230 (35.6)	0.88
Media coverage				0.58
Excessive and overblown	298 (35.5)	81 (34.5)	217 (35.9)	
Accurate	407 (48.5)	120 (51.1)	287 (47.4)	
Not serious enough	135 (16.1)	34 (14.5)	101 (16.7)	
Media sources				0.65
International news on television	202 (26)	53 (24.9)	149 (26.4)	
National/local news on television	72 (0.3)	24 (11.3)	48 (8.5)	
International news on Internet	224 (28.8)	57 (26.8)	167 (29.6)	
National/local news on Internet	177 (22.8)	54 (23.4)	123 (21.8)	
Newspapers	28 (3.6)	6 (2.8)	22 (3.9)	
Social media	75 (9.6)	19 (8.9)	56 (9.9)	

Coping strategies were fairly consistent between those with and without previous epidemic experience. However, respondents with previous epidemic exposure reported using music as a coping strategy more frequently (43.9% vs. 33.7%, *p* = 0.004). Media coverage and sources of media did not differ by epidemic experience (*p* = 0.58 and *p* = 0.650). Media coverage was reported as “accurate” among 48.5% of all respondents and “overblown” by 35.5% of respondents (Table 2).

**Fig. 2:**
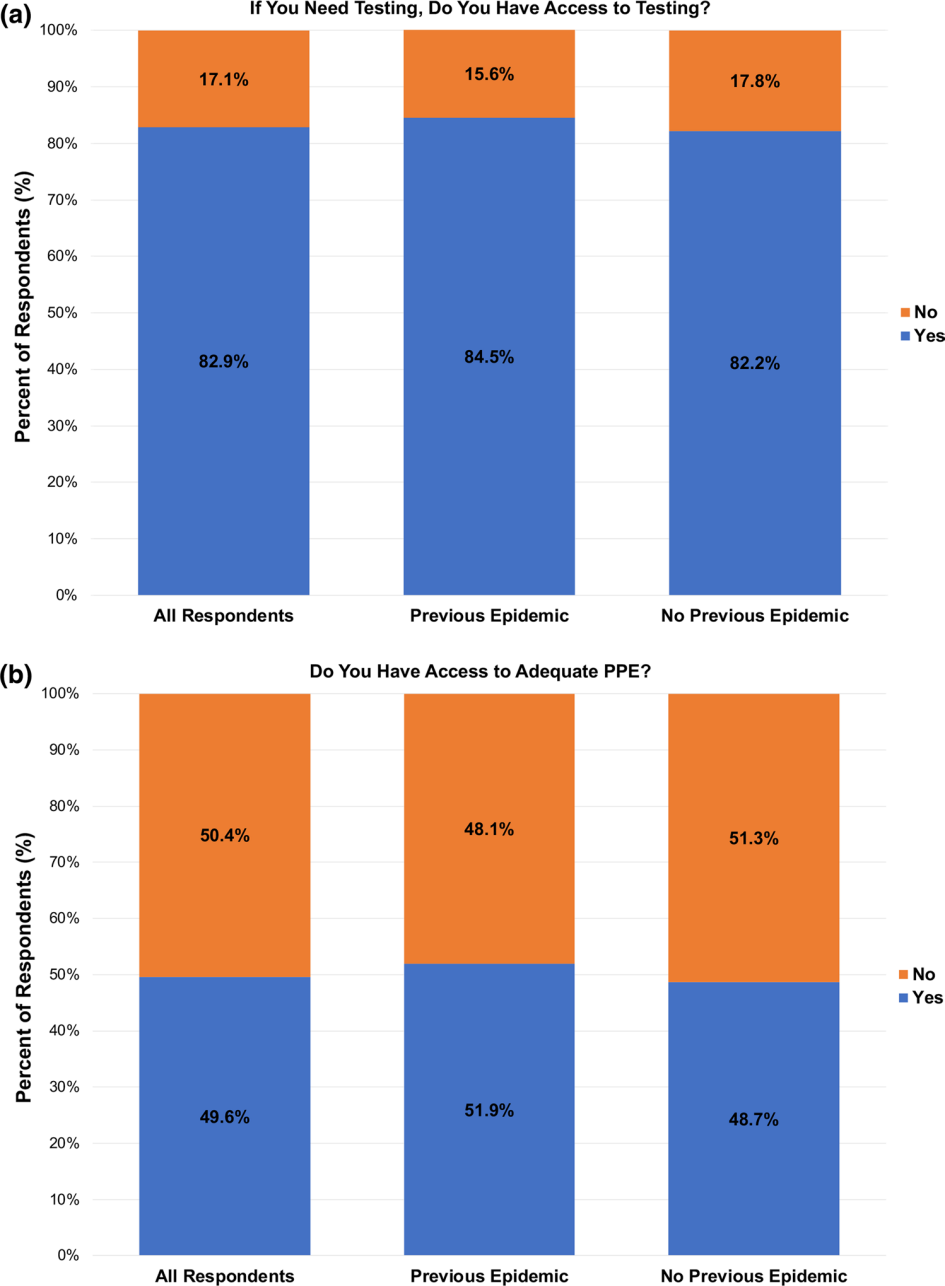
Impact of previous pandemics on COVID-19 preparedness **a** Bar graph comparing access to COVID-19 testing stratified by previous epidemic experience. **b** Bar graph comparing access to adequate PPE stratified by previous epidemic experience

Overall, 82.9% of respondents reported having access to COVID-19 testing. There was no difference in access to testing between surgeons with prior epidemic experience and those without epidemic experience (84.5% vs 82.2%, *p* = 0.440) (Fig. 2a). A total of 6.7% of surgeons reported being tested for COVID-19, without notable difference based on prior epidemic exposure (5.5% vs. 7.2%, *p* = 0.350). Formal hospital guidelines for epidemic/pandemic response were in place in 60.4% of respondents’ hospitals; prior epidemic experience did not impact on the presence of guidelines (64.2% vs. 59%, *p* = 0.190) (Table 3).

**Table 3:** COVID-19 preparedness stratified by previous epidemic experience

	All respondents (*n* = 902)	Previous epidemic experience (%) (*n* = 255)	No previous epidemic experience (%) (*n* = 647)	*p* value
Access COVID-19 testing				0.44
Yes	701 (82.9)	201 (84.5)	500 (82.2)	
No	145 (17.1)	37 (15.6)	108 (17.8)	
Personally tested for COVID-19				0.35
Yes	57 (6.7)	13 (5.5)	44 (7.2)	
No	789 (93.3)	225 (94.5)	564 (92.8)	
Formal hospital guidelines				0.19
Yes	452 (60.4)	131 (64.2)	321 (59.0)	
No	296 (39.6)	73 (35.8)	223 (41.0)	
Adequate PPE for frontline workers				0.4
Yes	415 (49.6)	121 (51.9)	294 (48.7)	
No	422 (50.4)	112 (48.1)	310 (51.3)	
Forms of PPE provided				
N-95 mask	451 (50.0)	130 (51.0)	321 (49.6)	0.71
Surgical mask	738 (81.8)	205 (80.4)	533 (82.4)	0.49
Face shield	415 (46.0)	113 (44.3)	302 (46.7)	0.52
Gown	491 (54.4)	131 (51.4)	360 (55.6)	0.25
Full-face respirator	95 (10.5)	29 (11.4)	66 (10.2)	0.61
Adequate ventilators				0.53
Yes	343 (41.0)	100 (42.7)	243 (40.4)	
No	493 (59.0)	134 (57.3)	359 (59.6)	

Surgeons reported having adequate PPE at a rate of 49.6%; prior epidemic experience did not impact PPE availability rates (51.9% vs. 48.7%, *p* = 0.400) (Fig. 2b). N95 masks were provided to 50% of respondents, surgical masks to 81.8%, face shields to 46%, gowns to 54.4%, and full-face respirators to 10.5%. There were no significant differences in the type of available PPE based on previous epidemic experience (*p* > 0.05). Surgeons reported that 41% of their hospitals had adequate ventilators for the volume of patients they expected (Table 3).

Of the 57 respondents who underwent viral testing, nine (15.8%) reported testing positive for COVID-19. In areas of prior epidemics, surgeons reported being placed into quarantine at a greater rate compared to those from areas without prior epidemics (28.6% vs. 20.7%, *p* = 0.014) (Table 4).

**Table 4:** COVID-19 response stratified by previous epidemic experience

	All respondents (*n* = 902)	Previous epidemic experience (%) (*n* = 255)	No previous epidemic experience (%) (*n* = 647)	*p* value
COVID-19 diagnosis				
Know someone diagnosed	392 (46.6)	104 (44.3)	288 (47.5)	0.39
Personally diagnosed	9 (1.1)	2 (0.8)	7 (1.2)	0.67
Personally quarantined				0.014
Yes	193 (22.9)	68 (28.6)	125 (20.7)	
No	649 (77.1)	170 (71.4)	479 (79.3)	
Hospital restrictions				
Quarantine upon return from travel	507 (56.2)	144 (56.5)	363 (56.1)	0.94
Limitations on domestic travel	483 (53.6)	130 (51)	353 (54.6)	0.33
Cancellation of academic activities	689 (76.4)	192 (75.3)	497 (76.8)	0.63
Nonessential staff to work from home	558 (61.9)	153 (60)	405 (62.6)	0.47
Cancellation of hospital meetings	674 (74.7)	183 (71.8)	491 (75.9)	0.2
Cancellation of elective surgeries	714 (79.2)	191 (74.9)	523 (80.8)	0.048
Government restrictions				
Cancel elective surgery	646 (71.6)	171 (67.1)	475 (73.4)	0.057
Shelter protection/self-isolation	570 (63.2)	148 (58.0)	422 (65.2)	0.044
No group gatherings > 50	365 (40.5)	106 (41.6)	259 (40.0)	0.67
No group gatherings > 100	488 (54.1)	125 (49.0)	363 (56.1)	0.055
Only gather with those in the same household	371 (41.1)	91 (35.7)	280 (42.3)	0.037
Closure of nonessential businesses	727 (80.6)	522 (80.7)	205 (80.4)	0.92
Closure of schools/universities	795 (88.1)	222 (87.1)	573 (88.6)	0.53
Closure of all dine-in restaurant opportunities	711 (78.8)	528 (81.6)	183 (71.8)	0.0011
Closure of public transportation	239 (26.5)	64 (25.1)	175 (27.1)	0.55
Restrictions on elderly for leaving home	426 (47.2)	116 (45.5)	310 (47.9)	0.51
Government stay-at-home order				0.058
Yes	688 (88.2)	182 (84.7)	506 (89.6)	
No	92 (11.8)	33 (15.4)	59 (10.4)	
Performing medical duties outside of specialty				0.98
Yes	183 (22.8)	51 (22.8)	132 (22.8)	
No	619 (77.2)	173 (77.2)	446 (77.2)	
Perception of government effectiveness				0.97
Appears in disarray/disorganized	88 (11.3)	23 (10.8)	65 (11.5)	
Taken some action but not enough	215 (27.6)	61 (28.5)	154 (27.3)	
Acceptable/appropriate	456 (58.5)	125 (58.4)	331 (58.6)	
Actions are excessive and unnecessary	20 (2.6)	5 (2.3)	15 (2.7)	
Perception of hospital effectiveness				0.53
Appears in disarray/disorganized	68 (8.8)	23 (10.8)	45 (8.0)	
Taken some action but not enough	215 (27.7)	56 (26.3)	159 (28.2)	
Acceptable/appropriate	477 (61.4)	128 (60.1)	349 (61.9)	
Actions are excessive and unnecessary	17 (2.2)	6 (2.8)	11 (2.0)	

**Fig. 3:**
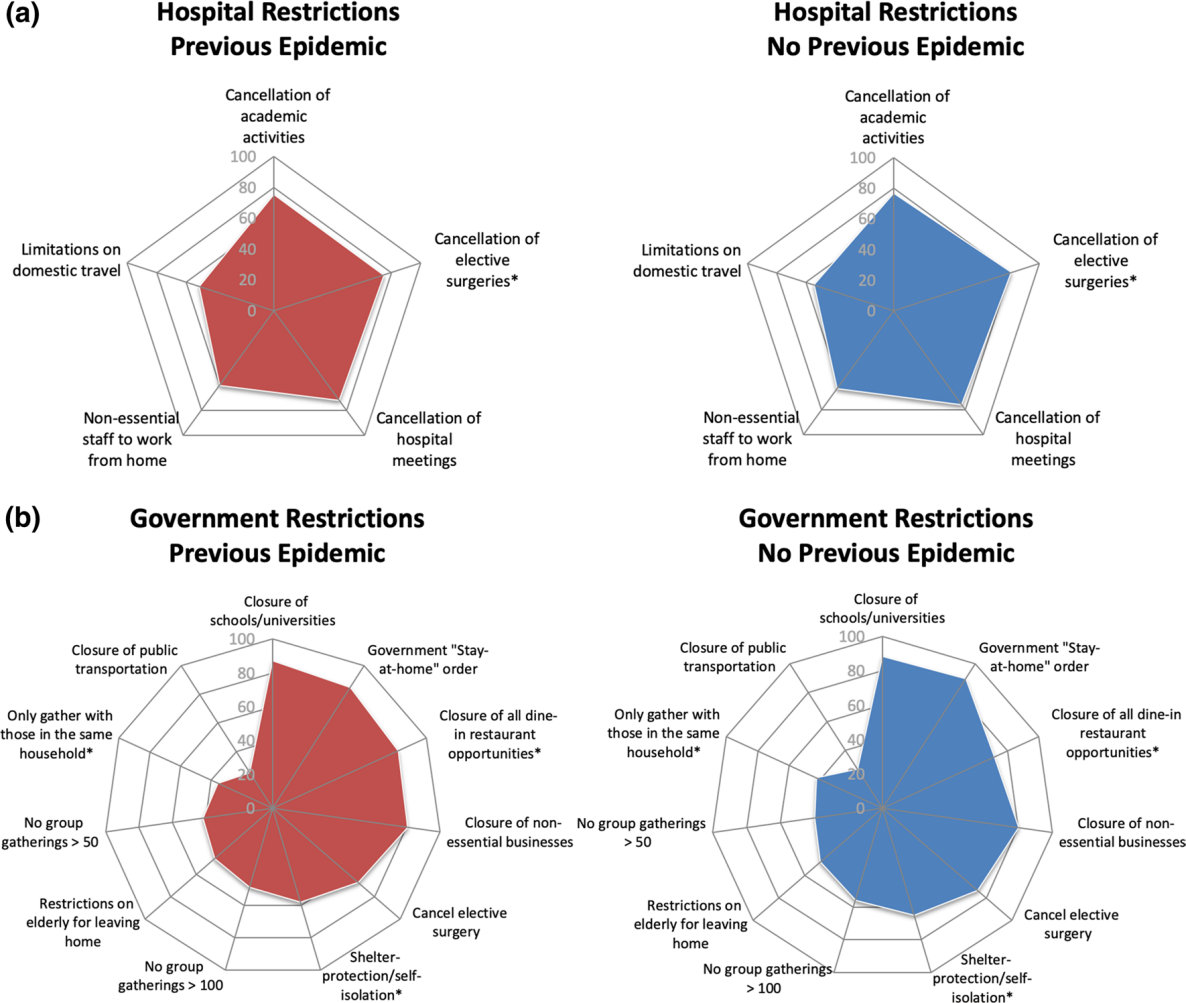
Radar chart depictions of current COVID-19 hospital and government policies by previous epidemic experience. Five-sided (pentagon) radar charts visually depicting cumulative percentage of responses verifying the enactment of a given COVID-19 **a** hospital and **b** government policy at the time of survey distribution. Queried policies are listed at the vertex of a given figure, whereby points falling on a vertex of the innermost pentagon correspond to a cumulative total of 0% of survey responses received. Each subsequent pentagon corresponds to a 20% increase in responses for a given category. *Indicates difference significant at the 95% confidence level (*P* < 0.05)

Respondents reported consistency among hospital restrictions; there were no differences in rates of quarantine after travel, domestic travel bans, cancellations of academic activities, work-from-home orders, or cancellation of hospital meetings (*p* > 0.05) (Fig. 3a). Surgeons from areas of prior epidemics reported a lower rate of hospital-mandated elective surgery cancellation compared to those from areas without prior epidemics (74.9% vs. 80.8%, *p* = 0.048). Respondents reported significant differences among government restrictions; governments with prior epidemic experience had fewer shelter-in-place orders (58% vs. 65.2%, *p* = 0.044), less restrictive bans on gathering with those outside their household (35.7% vs. 42.3%, *p* = 0.037), but more mandatory restaurant closures (81.6% vs. 71.8%, *p* = 0.001). There was no difference in government-mandated cancellation of elective surgeries (67.1% vs. 73.4%, *p* = 0.057) or stay-at-home orders (22.8% vs 22.8%, *p* = 0.98) (Table 4, Fig. 3b).

With regard to the impact of COVID-19 on the personal practice of surgeons, there were several significant differences between those with and without prior epidemic experience (Table 5). Respondents from areas with prior epidemics reported still performing elective spine surgery at a higher rate compared to those from areas without prior epidemics (26.1% vs. 15.6%, *p* < 0.001). However, there was no difference in the rate of essential spine surgeries or reduction in case volume (*p* > 0.05). There were significant differences in income lost (*p* = 0.004) and percent personal revenue lost (*p* = 0.042), with those from areas without epidemic experience reporting a larger financial impact from COVID-19. There were no significant differences in hospital revenue lost (*p* = 0.36), furloughs (*p* = 0.57), hospital layoffs (*p* = 0.41), personal layoffs (*p* = 0.38), or time frame to resume elective surgeries (*p* = 0.052) between surgeons with and without prior epidemic experience (Table 5).

**Table 5:** COVID-19 practice impact stratified by previous epidemic experience

	All respondents (*n* = 902)	Previous epidemic experience (%) (*n* = 255)	No previous epidemic experience (%) (*n* = 647)	*p* value
Performing elective spine surgery				0.0005
Yes	149 (18.5)	59 (26.1)	90 (15.6)	
No	655 (81.5)	167(73.9)	488 (84.4)	
Performing essential spine surgery				0.88
Yes	700 (87.3)	197 (87.6)	503 (87.2)	
No	102 (12.7)	28 (12.4)	74 (12.8)	
Percent cases cancelled/postponed per week				0.22
< 25%	72 (9.0)	24 (10.7)	48 (8.3)	
25–50%	69 (8.6)	19 (8.4)	50 (8.7)	
51–75%	123 (15.3)	42 (18.7)	81 (14.0)	
> 75%	539 (67.1)	140 (62.2)	399 (69.0)	
Top allocation of time				0.95
Spending time with family	312 (49.5)	90 (51.4)	222 (48.8)	
Personal wellness	59 (9.4)	18 (10.3)	41 (9.0)	
Resting	38 (6.0)	10 (5.7)	28 (6.2)	
Planning for future	19 (3.0)	6 (3.4)	13 (2.9)	
Engaging in hobbies	17 (2.7)	5 (2.9)	12 (2.6)	
Academic projects/research	32 (5.1)	6 (3.4)	26 (5.7)	
Community outreach programs	13 (2.1)	3 (1.7)	10 (2.2)	
Spine practice/medical center-related work	140 (22.2)	37 (21.1)	103 (22.6)	
Impact on income				0.004
Planned reduction, on salary	138 (18.1)	51 (24.5)	87 (15.7)	
No impact, on salary	244 (32.1)	62 (29.8)	182 (32.9)	
Planned reduction, compensation-based income	64 (8.4)	22 (10.6)	42 (7.6)	
No impact, compensation-based income	7 (0.9)	4 (1.9)	3 (0.5)	
Losing income	308 (40.5)	69 (33.2)	239 (43.2)	
Percent of personal revenue lost				0.042
< 25%	219 (28.9)	57 (27.5)	162 (29.5)	
25–50%	226 (29.9)	77 (37.2)	149 (27.1)	
51–75%	142 (18.8)	36 (17.4)	106 (19.3)	
> 75%	170 (22.5)	37 (17.9)	133 (24.2)	
Percent of hospital revenue lost				0.36
< 25%	169 (22.3)	47 (22.5)	122 (22.3)	
25–50%	199 (26.3)	64 (30.6)	135 (24.6)	
51–75%	207 (27.3)	53 (25.4)	154 (28.1)	
> 75%	182 (24.0)	45 (21.5)	137 (25.0)	
Staff furlough				0.57
Yes	307 (40.5)	91 (43.5)	216 (39.3)	
No	286 (37.7)	74 (35.4)	212 (38.6)	
Potentially	165 (21.8)	44 (21.1)	121 (22.0)	
Hospital layoffs				0.41
Yes	67 (8.8)	15 (7.2)	52 (9.4)	
No	586 (77.0)	160 (76.6)	426 (77.2)	
No, but have plans to	108 (14.2)	34 (16.3)	74 (13.4)	
Personally laid off staff				0.38
Yes	39 (5.1)	10 (4.8)	29 (5.2)	
No	683 (89.8)	191 (91.8)	492 (89.0)	
No, but have plans to	39 (5.1)	7 (3.4)	32 (5.8)	
Time frame to resume elective surgeries				0.052
No current stoppage	85 (10.6)	29 (13)	56 (9.7)	
< 2 weeks	31 (3.9)	12 (5.4)	19 (3.3)	
2–4 weeks	136 (16.9)	44 (19.6)	92 (15.9)	
1–2 months	127 (15.8)	40 (17.9)	87 (15.0)	
> 2 months	33 (4.1)	10 (4.5)	23 (4.0)	
Unknown time frame	392 (48.76)	89 (39.7)	303 (52.2)	
Timeline to resume “baseline operation”				0.38
< 2 weeks	96 (12.7)	23 (11.0)	73 (13.3)	
2–4 weeks	177 (23.3)	59 (28.2)	118 (21.5)	
4–6 weeks	177 (23.3)	44 (21.1)	133 (24.2)	
6–8 weeks	108 (14.2)	29 (13.9)	79 (14.4)	
8 + weeks	201 (26.5)	54 (25.8)	147 (26.7)	
Impact on how you treat patients in 1 year				
No change	133 (14.8)	36 (14.1)	97 (15.0)	0.74
Heightened awareness of hygiene	435 (48.2)	119 (46.7)	316 (48.8)	0.56
Will increase use of PPE	344 (38.1)	94 (36.9)	250 (38.6)	0.62
Ask patient to reschedule if they feel sick	285 (31.6)	86 (3.7)	199 (30.8)	0.39
Pursue increased non-operative measures prior to surgery	150 (16.6)	43 (16.9)	107 (16.5)	0.96
Growth in digital options for communication	314 (34.8)	77 (30.2)	237 (36.6)	0.068

Multivariate regression analysis, controlling for statistically significant demographic differences (geographic region, population, fellowship training, and practice breakdown), revealed that prior epidemic exposure was independently associated with an increase in respondents reporting personal health as a source of stress (OR 1.66; 95% CI 1.21–2.27; *p* = 0.0015), music as a coping strategy (OR 1.67; 95% CI 1.21–2.30; *p* < 0.001, and still performing elective spine surgery (OR 1.55; 95% CI 1.01–2.38; *p* = 0.0035). The differences in hospital cancellations of elective surgeries (*p* = 0.960), government-mandated shelter-in-place orders (*p* = 0.290), bans on gathering with those outside their household (*p* = 0.710), and mandatory restaurant closures (*p* = 0.760) on univariate analysis were not significant after multivariate analysis. Prior epidemic exposure was also independently associated with respondents reporting impact on income (LR 12.70, *p* = 0.012) and personal revenue lost (LR 9.62, *p* = 0.022) (Table 6).

**Table 6:** Multivariable analysis of impact on prior epidemic exposure

Variable	Prior epidemic exposure odds ratio (95% CI)	*p* value
Greatest stressors		
Personal health	1.66 (1.21–2.27)	0.0015
Coping mechanisms for stress		
Music	1.67 (1.21–2.3)	0.0016
Has been personally quarantined	1.24 (0.83–1.83)	0.29
Hospital Restrictions		
Cancellation of elective surgeries	0.99 (0.67–1.46)	0.96
Government restrictions		
Shelter protection/self-isolation	0.84 (0.61–1.16)	0.29
Only gather with those in the same household	0.94 (0.68–1.3)	0.71
Closure of all dine-in restaurant opportunities	0.76 (0.52–1.11)	0.76
Performing elective spine surgery	1.55 (1.01–2.38)	0.045

Variable	Prior epidemic exposure likelihood ratio	*p* value
Impact on income	12.79	0.012
Percent of personal revenue lost	9.62	0.022

Multivariate logistic regression analysis of effect of previous epidemic exposure on current response and preparedness controlling for differences in baseline demographics (home city population, region, fellowship training, and percent research). Variables with *p* > 0.2 from univariate analysis were tested. Odds ratios with 95% confidence interval reported for dichotomous categorical variables; likelihood ratios reported for variables with multiple responses

**Fig. 4:**
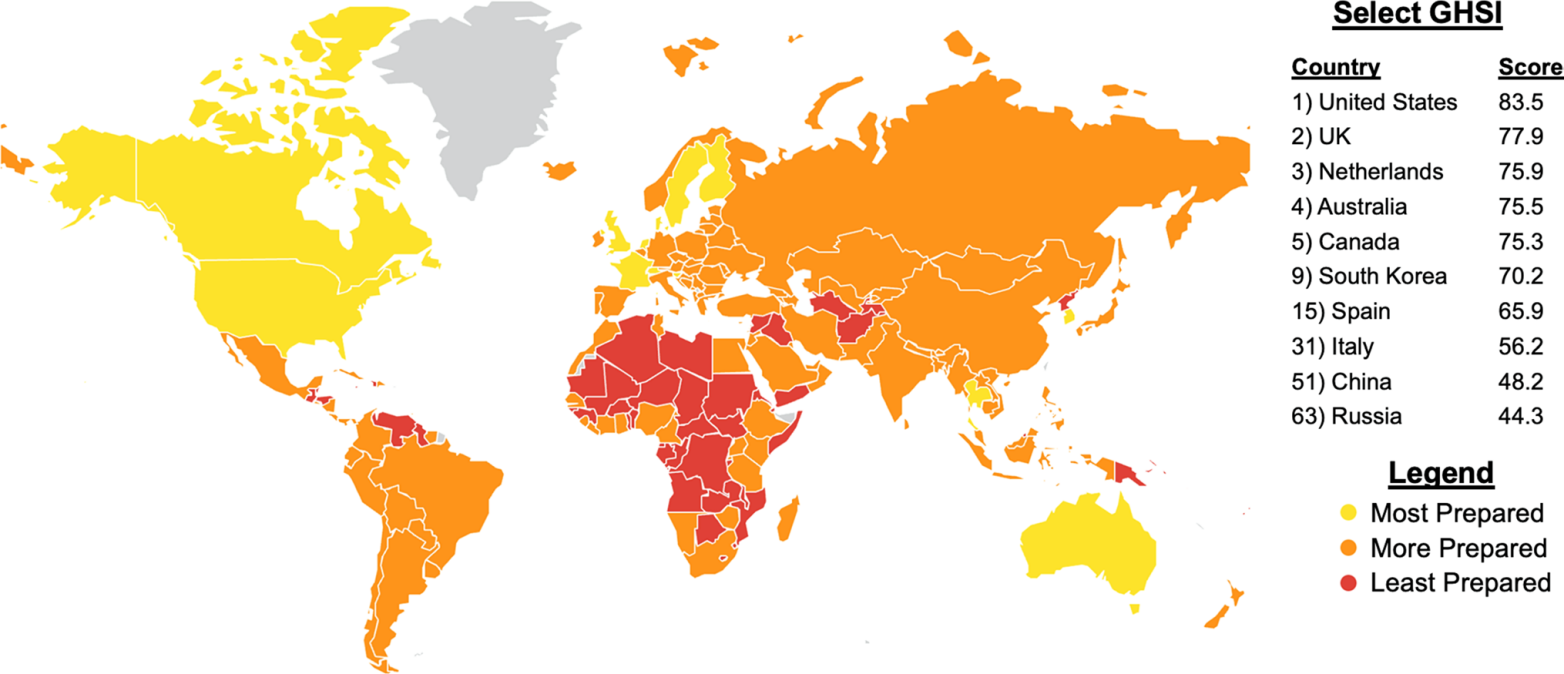
Global health security index scores by country. World map depicting Global Health Security Index scores by country. Color of countries indicate relative preparedness for global pandemic as ranked by Johns Hopkins Center for Health Security. Red, least prepared; Orange, more prepared; Yellow, most prepared; average overall GHSI score is 40.2. Data source: Nuclear Threat Initiative and Johns Hopkins Center for Health Security

**Fig. 5:**
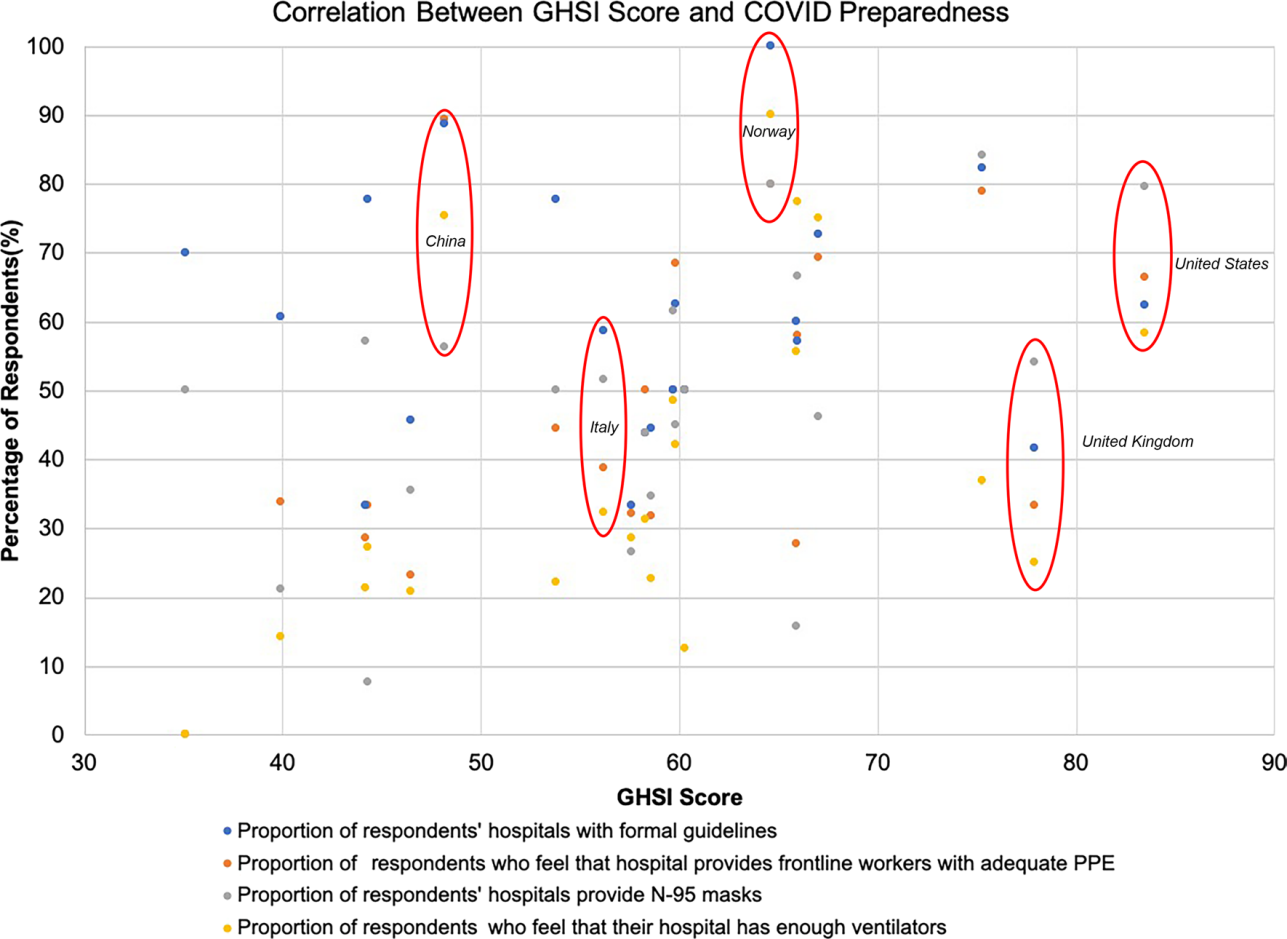
COVID-19 preparedness perceptions and global health security index scores. Scatter plot of COVID-19 preparedness perceptions and Global Health Security Index scores. All countries with > 10 respondents were included in the analysis (*n* = 687). A total of 21 countries were included. Mean responses to questions on the presence of formal guidelines, adequate PPE, N95 masks, and ventilators were plotted against the GHSI score of respondents’ countries. Linear regression analysis revealed poor correlations with *R*^2^ < 0.3

**Fig. 6:**
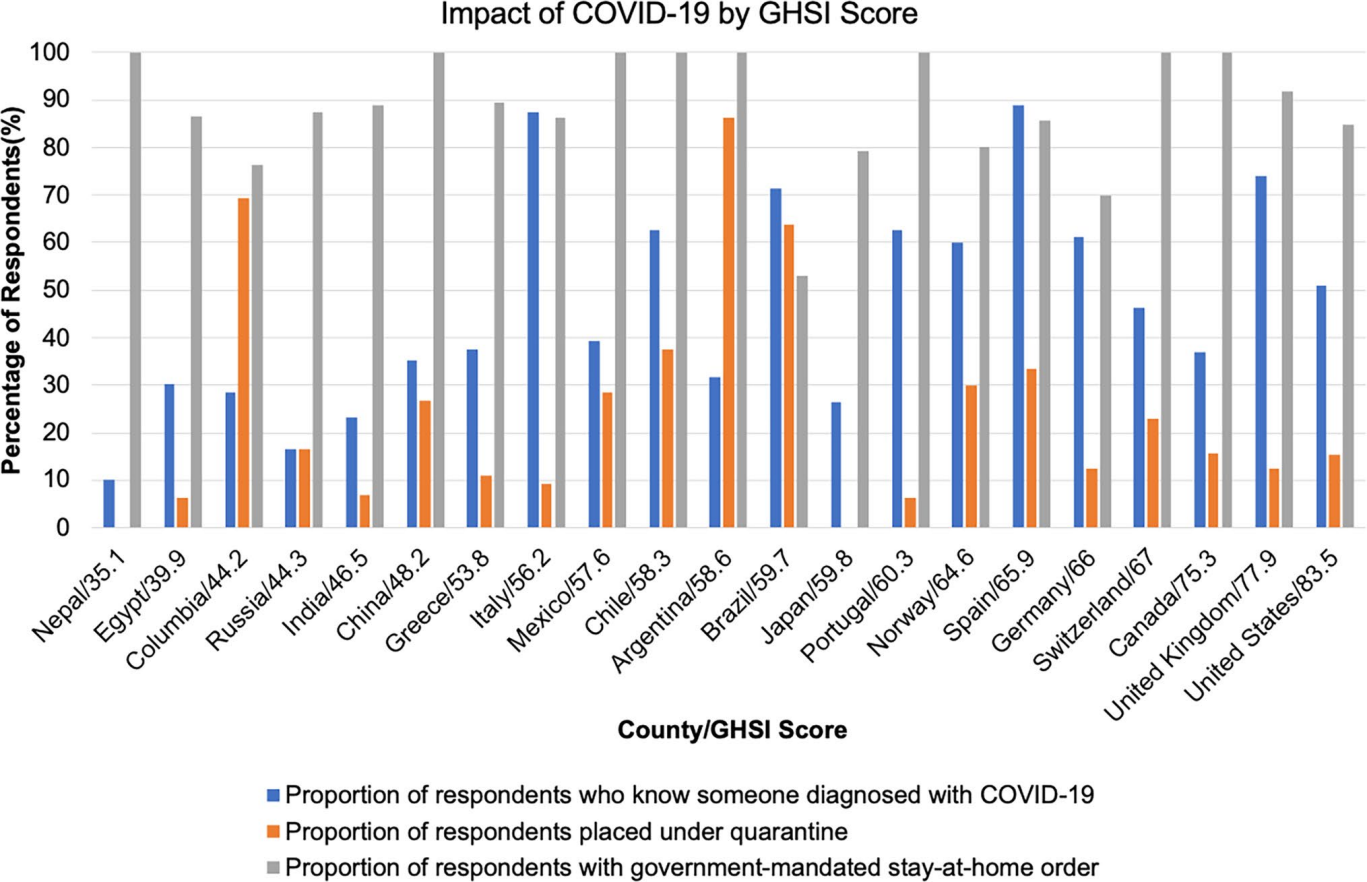
Impact of COVID-19 by GHSI score. Bar graph comparing the impact of COVID-19 stratified by country/GHSI score. All countries with > 10 respondents were included in the analysis (*n* = 687). A total of 21 countries were included

**Fig. 7:**
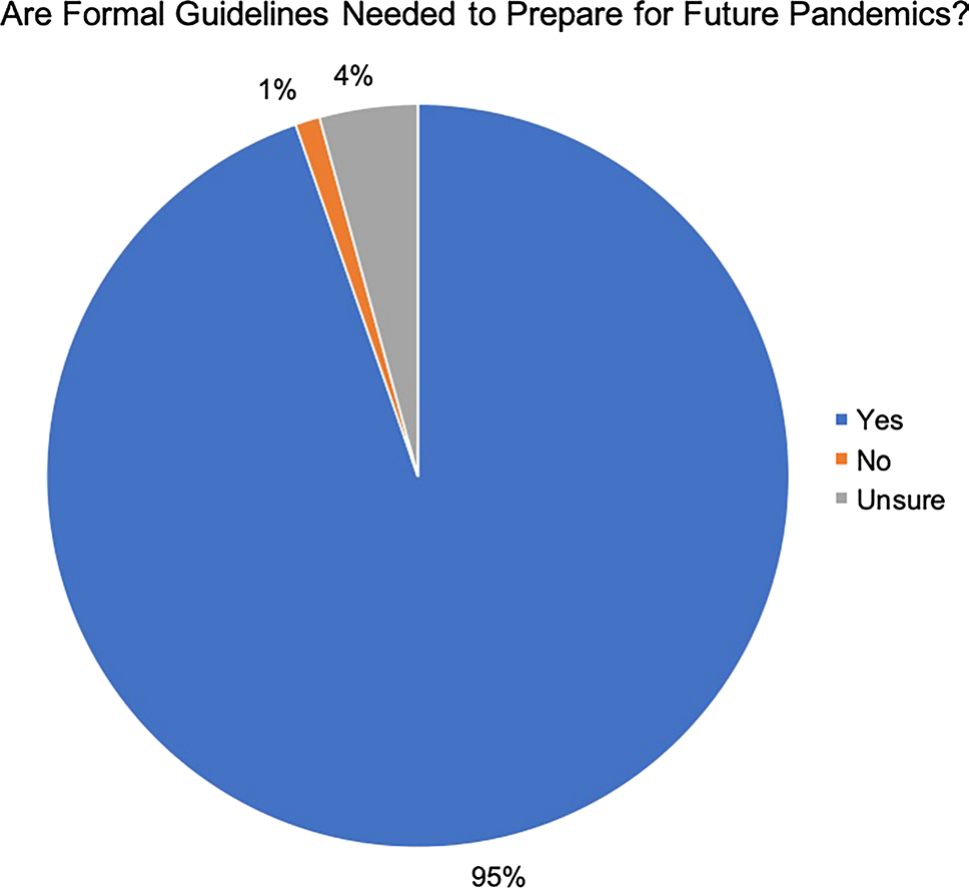
Assessing the need for formal international guidelines. Pie chart reporting the overwhelming support for international formal guidelines to mitigate the impact of future pandemics. 95% of respondents from all regions of the world were in favor of formal guidelines

Global Health Security Index scores for countries with significant burdens of COVID-19 were reported (Fig. 4). The USA received a GHSI score of 83.5 and was ranked first, as the most prepared country for a global pandemic. There was poor correlation between GHSI score and access to adequate PPE (*R*^2^ = 0.26, *p* = 0.019), access to N95 masks (*R*^2^ = 0.26, *p* = 0.019), and access to adequate ventilators (*R*^2^ = 0.23, *p* = 0.029). There was no correlation between GHSI score and formal hospital guidelines (*R*^2^ = 0.0004, *p* = 0.930) (Fig. 5). The impact of COVID-19 and government responses varied greatly among countries, regardless of GHSI score (Fig. 6). Overall, 95% of surgeons felt that future formal guidelines are needed to mitigate future pandemics (Fig. 7).

## Discussion

COVID-19 is a defining global health crisis. Understanding how spine surgeons around the world prepared for, and responded to, COVID-19 will help guide response to future pandemics. Louie et al. [27] highlighted, in over 900 spine surgeons worldwide, that COVID-19 had a substantial impact upon their patient care, practice, and personal lives; however, such impact varied. As such, our goal was to outline whether previous experience with outbreaks/pandemics played a role in surgeons’ preparedness, response, and perceptions. Interestingly, based on survey results, surgeons felt generally underprepared for a pandemic of this magnitude. The WHO and other global health experts have prioritized learning from previous health crises, yet our study noted that regions with previous infectious disease outbreaks were no more prepared to respond to COVID-19. Our study further outlines that previous measures of global health security were not predictive of preparedness or minimized impact.

### Impact of previous epidemics on preparedness

The COVID-19 pandemic is neither novel nor unexpected. During the twentieth century, there were three major pandemics that ravaged the globe: the H1N1 Spanish flu of 1918, the H2N2 Asian flu of 1957, and the H3N2 Hong Kong flu of 1968 [37]. Since 1968, only the HIV/AIDs outbreak spread across the globe and has had widespread impact on healthcare workers [38]. The more recent epidemic level outbreaks of SARS in 2002, H1N1 Swine flu in 2009, MERS in 2012, and Ebola in 2013 provided certain regions around the world with an early opportunity to prepare for deadly infectious disease outbreaks [11, 12, 17, 18, 39–41].

Our survey indicates that respondents who indicated prior experience with the SARS, MERS, H1N1, and Ebola outbreaks were no better prepared to take on the COVID-19 pandemic. This likely indicates that countries around the world have struggled to change government and hospital policy based upon prior experiences. Limitations in access and availability of testing have been cited as a major shortcoming in the media [42, 43]. Our results indicate that access to testing is no longer a major limitation for surgeons, with over 82% of surgeons reporting access to a COVID-19 test. However, only 6.7% of surgeons around the world reported actually being tested. Surprisingly, 5.5% of respondents with prior epidemic experience reported being tested compared to 7.2% of those without prior epidemic experience. This gap between testing availability and completed testing indicates that universal testing of healthcare workers is not occurring.

Numerous health departments across the world have outlined that formal local and institutional guidelines are critical for pandemic preparedness [44–46]. In 2009, the WHO reported that many countries around the world were in the process of forming a pandemic plan, but no standard pattern in content or timing was in place, and many countries were waiting for WHO to lead with their own plan. The WHO warned that without regional or global leadership on formal pandemic plans, preparedness could diverge even further across the world [46]. In our study, a surprisingly low 60.4% of respondents reported that formal hospital guidelines for pandemic response were in place. This number only marginally increased to 64.2% among respondents with prior epidemic exposure but did not reach statistical significance. Clearly, formal institutional guidelines should have been a priority among all hospitals prior to the outbreak reaching pandemic proportions.

Another preparedness deficiency was access to personal protective equipment and other critical hospital resources. The media in the USA and across the world highlighted the critical lack of PPE that frontline healthcare workers faced in the early days of the COVID-19 outbreak [7, 8, 47–49]. While many respondents felt the media was sensationalizing the outbreak, our study indicates that the critical shortage of PPE is real with only 49.6% of respondents reporting access to adequate PPE. Even in regions with prior health crises, the availability of PPE was not significantly improved. Another key resource limitation facing health systems during this pandemic is the ventilator shortage. Not only are physicians facing the possibility of difficult decisions surrounding allocation of ventilators [50, 51], but operating room anesthesia machines are being reallocated to intensive care units (ICUs) closing operating rooms (ORs) for surgeon use [52]. An alarmingly low 41% of respondents reported adequate ventilator supplies, and access to ventilators was not improved by experience with prior epidemics. Clearly, a need exists for larger stockpiles of these critical resources that can be mobilized during global pandemics.

Overall, respondents from countries with previous infectious disease outbreaks did not report improved government or hospital-level preparedness. This indicates that health systems and governments likely failed to learn from prior health crises or did not dedicate the time, resources, or manpower to strategic planning. Regardless of these prior oversights, there is now a major need to come together and prepare for future pandemics.

### Impact of previous epidemics on COVID response

In the early days of COVID-19, there were a variety of responses to the growing threat spreading across the world. China instituted a swift government-mandated lockdown of Wuhan in the Hubei Province in an attempt to slow the spread [53–55]. South Korea quickly implemented a widespread testing initiative that helped to isolate cases and prevent a prolonged nationwide lockdown [56, 57]. Both of these nations had previous experience with SARS, MERS, and other infectious disease outbreaks and had instituted national policies allowing for rapid approval of testing in the face of new disease outbreaks [58]. Prior experiences likely guided the response to, and impact of, COVID-19.

Surprisingly, the government and hospital restrictions instituted around the world were fairly consistent. Respondents reported high rates of government-mandated cancellation of elective surgeries, mandatory stay-at-home orders, limitations on group gatherings, and closure of businesses and schools. A few subtle differences were noted. Respondents who indicated prior experience with infectious disease epidemics reported being placed into quarantine at a higher rate after exposure to COVID-19. This may be because these governments had prior experience with quarantines and were willing to swiftly institute mandatory isolation.

Hospital-based restrictions were also remarkably conserved across the world. There were high rates of travel bans, cancellations of academic activities, cancellations of hospital meetings, and work-from-home orders. Interestingly, prior epidemic experience was an independent predictor of still performing elective spine surgeries. The significance of this finding is unclear, given that epidemic experience was not predictive of preparedness.

Overall, surgeons appear to be somewhat dissatisfied with their governmental and hospital responses. A total of 58.5% of respondents reported their government’s response as “acceptable,” while 27.6% rate their government’s action as “not enough.” Satisfaction rates with hospital responses are similar with 61.4% of respondents rating their hospital’s response as “acceptable,” while 27.7% rate their hospital’s action as “not enough.” Respondents with prior infectious disease epidemic experience did not rate their government or hospital response any better. A moral and ethical obligation exists to improve our ability to respond to future crises.

### COVID-19 and spine practice across the world

Government and hospital policies in response to COVID-19 are impacting spine practices across the world. Over 67% of respondents reported a greater than 75% decrease in their weekly case volume. This reduction in volume has led to significant economic concerns among surgeons [59]. Nearly 70% of surgeons reported a reduction in income from the current COVID-19 crisis. However, having prior experience with epidemics did lead to a significant decrease in rates of reported income loss. This may be confounded by the fact that most countries with prior epidemics utilize government run health systems.

Apart from economically impacting surgeons, the COVID-19 pandemic has financial implications for all healthcare staff. In this study, 40.5% of respondents reported having staff furlough at their institutions, with 8.8% reporting layoffs. Unfortunately, having prior experience with infectious disease epidemics did not protect against these financial effects. This point highlights the need for comprehensive government policies that prevent these economic impacts, rather than reacting to them.

### Does the global health security index accurately predict impact of COVID-19?

The GHSI is the first comprehensive assessment of health security and pandemic preparedness across 195 countries [20]. The GHSI provides a ranking by overall pandemic preparedness, early detection capabilities, and ability to mitigate a health disaster. The goal of the GHSI project was to use data obtained from prior disease outbreaks to improve the international capability to address pandemics [21]. Our survey results indicate that the GHSI was poorly correlated with COVID-19 preparedness and surgeons’ perceptions on response. Countries such as the USA were rated as “most prepared” by the GHSI yet were not adequately prepared based on our survey. China, a country rated as “more prepared” with a low GHSI of 48.2, had similar access to PPE and critical resources as the USA.

The poor performance of the GHSI may indicate that traditional methods for assessing pandemic preparedness are faulty, or COVID-19 did not follow the patterns established by previous infectious disease outbreaks. Either way, we have an ethical and moral obligation to learn from the current situation to revamp the ways in which we prepare for pandemics and the way we assess pandemic preparedness. Improvements in global coordination and cooperation have the potential to lessen the impact of infectious disease outbreaks, not only on surgeons, but on all of humanity.

### Limitations

As with many questionnaire-based studies, there are limitations to this study. The survey distribution was limited to the current AO Spine surgeon members network. The survey was sent out to 3805 spine surgeons worldwide; however, only 902 surgeons responded (23.7%). This may introduce a response bias because individuals with strong opinions may be more likely to respond. Previous studies have described that low response rate is a risk factor for low validity, but does not necessitate low validity [60]. Response rates are important to consider, but, independently, should not be considered a proxy for study validity.

Our study lacked the power to break down responses by individual country. Therefore, certain countries may have adequately learned from previous epidemics, but their response is diluted by the many others who did not. We attempted to control for this by using geographic region in our multivariate analysis. However, there may be questionable generalizability in regions in which there were few or no respondents. The timing of the survey may have also impacted our results as countries around the world were at different stages of the pandemic when they received the questionnaire. Given the limit of survey length due to fatigue, we were not able to explore all of the possible domains related to COVID-19.

Finally, our targeted demographic was AO spine surgeons. This is one group of subspecialty surgeons, and the results may not represent the view and concerns of other medical specialties. However, given that COVID-19 is impacting all healthcare providers around the world, we feel spine surgeons are reasonably representative of other surgical providers. We are unable to comment on COVID-19 preparedness or impact for the general public. Despite these limitations, this survey remains the largest, international effort to assess multiple domains of the impact of COVID-19 on spine surgeons.

## Conclusion

This is the first, international study to assess the impact of COVID-19 on spine surgeons in an effort to explore the effect of previous epidemics on preparedness and response. This study outlines that previous infectious disease outbreaks had only subtle influence on the impact of COVID-19 and no substantial bearing on preparation for the current pandemic. Furthermore, current methods for assessing preparedness, such as GHSI, were poorly correlated with preparedness for the current outbreak. Findings from our study indicate that COVID-19 substantially impacted spine surgeons globally; therefore, we have a moral obligation to help lead the charge in developing comprehensive policies to mitigate the impact of this current, and any future, public health crises.
